# Data article on institutional framework and business survivals of informal entrepreneurs in electronics market, Southwest, Nigeria

**DOI:** 10.1016/j.dib.2018.05.092

**Published:** 2018-05-24

**Authors:** Ayeni Adebanji, Olaleke Oluseye Ogunnaike, O.O. Iyiola, Omotola Adedoyin Ezenwoke, Stephen Ibidunni

**Affiliations:** a Landmark University, Nigeria; b Covenant University, Nigeria

**Keywords:** Intuitional framework, Business survivals, Informal entrepreneurs, Electronics market, Nigeria

## Abstract

The article presented data on how institutional framework has provided opportunities for the survival of informal entrepreneurs. In other words, data were gathered based on how the informal entrepreneurs in the electronics market were able to manipulate or avoid law enforcement officials thereby leading to sustainable business performance. Emphasis was laid on four southwestern states in Nigeria to determine the survivals of informal business. Data were gathered based on descriptive survey research design. The population of this study included all informal entrepreneurs in some selected electronics markets. Mean score and standard deviation were used as statistical tools for data presentation. The field data set is made widely accessible to enable critical or a more comprehensive investigation.

**Specification Table**

**Table:** 

**Subject area**	Business, Management
**More Specific Subject Area**	Business and Informal Entrepreneurship
**Type of Data**	Table
**How Data was Acquired**	Researcher-made questionnaire analysis
**Data format**	Raw, analyzed, data
**Experimental Factors**	Sample consisted of electronics informal entrepreneur in Nigeria. The researcher-made questionnaire which contained data on opportunity exploitation of institutional frameworks (avoidance and manipulative ability) and business survival electronics informal entrepreneurs
**Experimental features**	Opportunity exploitation of institutional frameworks facilitators are one of the factors endangering the growth of informal businesses while being seen as a vice in the society
**Data source location**	South west Nigeria
**Data Accessibility**	Data is included in this article

**Value of data**

• These data present descriptive data on opportunity exploitation of institutional frameworks by informal entrepreneurs in electronics markets as it relates to the survival of their business. This is geared towards the stability and possible recognition of business activities of informal entrepreneur.
• The results showed that the use of avoidance and manipulative skills by the entrepreneurs on the regulatory agencies was the bases of engaging in the legal business activities informally. Practical involvement of informal entrepreneurs in framing business laws will be very helpful for employment generation. The results of this study can be used to improve policy creation and drive towards business growth and sustainability.

## 1 Introduction

Extant studies on informal entrepreneurship considered motivation of informal entrepreneurship from the standpoint of institutions [3], [4]. Some entrepreneurs are involved in informal market as a result of some loopholes within the institutional framework also known as opportunity exploitation facilitators. In order words, conflicting laws and policies within the Nigerian business environment, which is further enhanced by bribery and corruption [4]. Corruption is an act which deviates from the formal rules of conduct governing the action of someone in the position of public authority because of private motive such as wealth, power and status. This data article gathered data on how opportunity exploitation facilitators have contributed to rising activities in the informal economy.

## 2 Data

As shown in Table 1 below, a total of three hundred and twenty copies of questionnaire were administered to the informal entrepreneurs of four selected informal electronics markets in southwest Nigeria. It was distributed equally to all the different electronics informal markets in those locations. All the distributed questionnaires were retrieved, which amounted to a 100% response rate. This was attributed to using the gatekeeper method in accessing the population [1]. The gatekeeper method of data gathering was adopted because it provides the researcher with a person who works amongst the intended respondents and has a good relation or highly placed with authority in distribution and data gathering [1], [2].

**Table 1:** Response rate of copies of questionnaire administered.

Questionnaire	Number of respondents	Response rate (%)
Returned	320	100
Not Returned	Nil	0
Total	320	100

Based on the copies of questionnaire retrieved, below is the demographic information showing the distribution based on age gender and educational qualification on Table 2.

**Table 2:** Distribution of biographical data of the respondents.

Demographic variables		State 1		State 2		State 3		State 4		Total	Percentage
		Freq	%	Freq	%	Freq	%	Freq	%		%
Gender:	M	40	12.5	56	17.5	70	21.9	57	17.8	223	69.7
	F	40	12.5	24	7.5	10	3.1	23	7.2	97	30.3
Age:	Under 25	34	10.6	14	4.4	21	6.6	21	6.6	90	28.1
	26–35 years	27	8.4	25	7.8	32	10	25	7.8	109	34.1
	36–45 years	13	4.1	22	6.9	17	5.3	22	6.9	74	23.1
	46 years and above	6	1.9	19	5.9	10	3.1	12	3.8	47	14.7
Marital Status	Single	39	12.2	29	9.1	42	13.1	34	10.6	144	43
	Married	30	9.4	33	10.3	31	9.7	33	10.3	127	39.7
	Others	11	3.4	18	5.6	7	2.2	13	4.1	49	15.3
Educational Qualification	Others	0	0	1	0.3	1	0.3	1	0.3	3	0.9
	MSc/MBA/M.Ed	1	0.3	0	0	0	0	1	0.3	2	0.6
	HND/BSc.	22	34.9	8	5.6	3	1.6	5	3.0	58	18.1
	OND/NCE	18	5.6	16	5.0	15	4.7	21	6.6	70	21.9
	SSCE	42	13.1	28	8.8	37	11.6	27	8.4	134	41.9
	Pri.Sch. Certificate	13	4.1	11	3.4	4	1.3	6	1.9	34	10.6
	No Formal Education	2	0.6	5	1.6	3	0.9	9	2.8	19	5.9

### 2.1 Gender distribution

Table 2 above shows the frequency distribution of respondents’ demographic data. The distribution of gender reveals that male respondents were 223(69.7%) and female respondents were 97 (30.3%). Despite the 39.3% difference between the two genders, data obtained represents a rich opinion of both genders. State 3 had the highest number of male respondents (70) representing 21.9% of the total number of male respondents and State 1 had the lowest number of male respondents (40) representing 12.5% of the total number of male respondents. On the other hand, State 1 had the highest number of female respondents (40) representing 12.5% of the total number of female respondents and state 3 had the lowest number of female respondents (10) representing 3.1% of the total number of female respondents. This validates the uneven distribution of respondents based on gender in the informal electronics market.

### 2.2 Age distribution

The age distribution revealed that 90 (28.1%) were respondents between ages under 25 years, 109 (34.1%) were respondents between ages 26–34 years, 74 (23.1%) were respondents 36–45 years and 47 (14.7%) were respondents 46 years and above. The result indicates that most of the respondents were between the ages 26–35 years (109) representing 34.1% of the total number of respondents. However, State 3 had the highest respondent of 32 between the ages 26–35 representing 10%. Respondents within the age bracket above 46 years were the minority, with state 1 having the lowest number of respondents in this age bracket (6) representing 1.9% of the total number of respondents. This implies that most respondents undertaking the informal entrepreneurship in the electronics market in southwest Nigeria, are mostly between the ages 26–35 years. This also shows that most of the respondents are young adults who aside have the youthful energy for such a laborious task are equipped with an inner drive (in some cases, called entrepreneurial spirit) to make lawful earnings.

### 2.3 Degree programme attained

Information provided by respondents in Table 2 on degree programme attained by the respondents shows that 134 (41.9%) were SSCE holders, 70 (21.9%) had OND/NCE, 58 (18.1%) attained HND/B.sc, 34 (10.6%) were Primary school certificate holders, 19 (5.9%), respondents who attained other certifications were 3 (0.9%), 19 (5.9%) had no formal education and 2 (0.6%) were M.Sc/MBA/M.Ed. The degree programme results revealed that more of the respondents were SSCE certificate holders (134) followed by OND/NCE holder, 70 and the least was MSc/MBA/M.Ed certificate holders (2) However, the distribution of degree programme attainment of respondents cuts across the different levels of certification, which implies that the opinions of respondents from different levels of certification were considered. It also depicted that there is a progression in the informal market in attaining knowledge while being hindered by certain undefined circumstances.

### 2.4 Marital status

Information provided by respondents in Table 2 on marital status by the respondents shows that 144 (43%) were single, 127 (39.7%) were married and other who were neither single or married were 49 (15.3%). The marital status revealed that more of the respondents were singles (144) followed by those who are married, 127 and the least was others (49). However, the distribution of marital status attainment of respondents displayed a high level of responsibility and sacrifice with the attainment of 127 (39.7%) married respondent and 144 (43%) marital potentials. It further displays the high level of commitment for another individual provision coupled with engaging in such a risky activity.

Table 3 above reveals that when respondents were asked if they operated in market because of the ease to spot threat, most of the respondents answered positively to the statement. The analysis in the table shows that the mean scores of the respondents from state 1, 2, 3, and 4, are 3.3625, 2.6625.

**Table 3:** Descriptive statistics of items measuring entrepreneurship institutional framework and business survivals.

**Statement**	**State 1**		**State 2**		**State 3**		**State 4**		**Total**	
	**Mean**	**Standard deviation**	**Mean**	**Standard deviation**	**Mean**	**Standard deviation**	**Mean**	**Standard deviation**	**Mean**	**Standard deviation**
Operation in market because easy to spot threat	3.3625	1.03425	2.6625	1.16862	3.1625	1.14122	3.0875	1.29452	3.0688	1.18571
Items sold are easy to escape with	3.1125	1.22209	2.6125	1.16373	3.0000	1.00631	2.9875	1.29745	2.9281	1.18685
Easy to escape penalties and punishment in informal business	3.0125	0.97427	2.5500	1.16814	3.0250	1.03085	2.9375	1.21534	2.8812	1.11345
Avoided tax helps me to operate here	2.9750	1.05513	2.4625	1.12445	2.9125	0.94392	3.1125	1.26285	2.8656	1.12429
Easy to embark on implicit negotiation with law enforcement	2.9875	1.21690	2.5000	1.21176	2.7750	0.96751	2.9375	1.29599	2.8000	1.18929
I will continue to operate in the informal market because I can easily bribe law enforcement agency	3.2750	1.05513	2.5000	1.25284	2.7250	0.91368	3.0375	1.31634	2.8844	1.17807
Adjustment of the Nigerian law by employing below 18years in informal business	3.1750	1.17759	2.1125	1.19061	2.9125	1.13844	2.6500	1.46780	2.7125	1.30534
Changes from the technology environment makes me us relevant	3.2875	0.98333	3.0000	1.09081	3.3125	0.83581	3.2125	1.12164	3.2031	1.01678
Speedy answering of customers because of the threat in the business operation	3.1250	1.14045	3.7750	1.28255	3.5000	0.94132	4.0125	1.07319	3.6031	1.15912
The speed I deliver to my customers makes me better off than my competitors	3.1125	1.07907	4.6250	0.78555	4.2875	0.84485	4.1125	0.95459	4.0344	1.07788

3.1625 and 3.0875 respectively. On the other hand, respondents from state 1 and 3 agreed more promisingly to the statement with mean scores 3.3625 and 3.1625 respectively. This suggests that more respondents from state 1 and 3 opine that business operation in the informal market was mainly because of the ability of theirs in being able to spot threat (the lawful enforcement agency). The table shows that when respondents were asked if the products they choose to sell was based on the ease to escape with, most respondents favorably agreed to the statement. The analysis shows that the mean scores of the respondents from states 1, 2, 3, and 4 are 3.1125, 2.6125, 3.0000 and 2.9875 respectively. This implies that more informal entrepreneurs from state 1 and 3 with mean scores of 3.1125 and 3.0000 believe that it is required to sell items that can easily be carted with when the need to escape of enforcement agency arises.

The table also reveals that most respondents affirmed that the engagement in informal business comes as a result of escaping penalties and punishment of engaging in business activity informally from the enforcement agencies. The analysis revealed that the mean scores for state 1, 2, 3, and 4 are 3.0125, 2.5500, 3.0250 and 2.9375 respectively. This result suggests that more respondents from state 1 and 3 with mean scores 3.0125 and 3.0250 respectively, are of the opinion that informal entrepreneurs have developed methods of escaping penalties and mutable punishment while engaging in informal business practices. The table reveals that respondents affirmed that the avoided tax payment creates an avenue for operating in the informal business with evidence from state 4 being the highest, showing a mean of 3.1125 with other states mean reflecting 2.9750, 2.4625 and 2.9125 for state 1, 2 and 3 respectively shows the negative skewed towards the statement. This could be related to the levies paid to the street urchins and use of bribe undertaken as tax in the different respondent׳s perspectives, thus reconciling a perceive high cost of running the business [3]. From the table, it clearly shows that embarking on implicit negotiation with law enforcements was not a criterion for engaging in the electronics business informally as the mean scores from the respondents reflected from states 1, 2 ,3 and 4 were 2.9875, 2.5000, 2.7750 and 2.9375 respectively. This displayed the negative skewness to the easiness of the law enforcement bypass in engaging in informal business practices, this asserting the findings of [4] in noting the necessity for a structure that must be enacted to absorb and encourage the informal entrepreneurs into the economic system.

The table displayed the ability to operate in the market comes as a result of easily bribing law enforcement agency was not totally supported. This was dully reflected in the high mean gotten from states 1 and 4 having 3.2750 and 3.0375 as their mean while states 2 and 3 displayed the downside to the statement with the evidence from of their mean being 2.5000 and 2.7250 respectively. This can only attest to the possibility of other factor(s) prompting as the reason for the business existence in the informal economy aside the entrepreneurs’ tenacity [3], [5], [6]. It was established from the table, that the finding of [7] with the mean score of 3.1750 from state 1 showed that employment in the informal business allows the positive possibility of engaging an individual below 18 a reality but states 2, 3 and 3 differ in this proposition with their mean score reflecting 2.1125, 2.9125 and 2.6500 respectively. Concurrently, the table displayed the informal entrepreneurs in the electronics business were relevant because of the constant changes in the technological industry with high mean from state 1, 2, 3 and 4 displaying 3.2875, 3.0000, 3.3125 and 3.2125 respectively. It was shown from the table that state 1 and 3 with the high mean of 3.2875 and 3.3125 were able to reflect the importance of the informal electronics market to the Nigeria society via the southwest study. Furthermore, it could be depicted that engaging in the electronics business, either sales or services involves lots of financial backing for the clients and business operators with the understanding of the dynamism in the technological sector [5], [8], [9]. The table reveals a high mean in the use of promptness while answering customers because of the impending threats accustomed to the business configuration of the informal economy. This was noticed in all the states with the highest being state 4 with 4.0125 while state 1, 2, and 3 had 3.1250, 3.7750 and 3.5000 respectively. This shows the need to engage in speed because of the elusiveness of security which comes from the security operatives in such markets. Finally, from the table, the speed of delivery in customer serve also serve as a weapon out besting competitors in the informal market and was relatively displayed with the high level of mean score in state 1, 2, 3 and 4 having 3.1125, 4.6250, 4.2875 and 4.1125 respectively. It was clearly displayed that state 2 and 4 with a very high mean of 4.6250 and 4.2875 clearly engaged this tactic in the informal electronics market to survive.

Findings from the descriptive statistics showed that some of the respondents agreed that the ability of theirs to avoid the payment of tax allows them to operate in the informal business while been able to be manipulate and evade the enforcement agencies vis-à-vis noting the technological changes in their business operation with the speed in responding to the customer׳s needs [3]. The descriptive statistics also revealed that most of the respondents clearly engage in manipulative and avoidance (via early deduction of the law enforcement agents) tactics to operate in the market with the necessity of high alertness as a prerequisite to survive in the market.

Based on the mean values above, a comprehensive analysis may likely be in consonance with the findings of [6], [10], [11]. These scholars suggested the need to redesign and incorporate the informal economy such that entrepreneurs operating in this sector, will be contributing to the economy as a whole as against the facet of parasitic existence. It may also confirm the findings of [5], [12] which shows the need for a consideration of informal entrepreneurs in the societal structure for economic development having the tendencies to affect employment and economic growth. Similar to many other studies in the field of management, the findings of this research is limited on the basis of generalization since a few states with unique characteristics were sampled for the study.

### 2.5 Study area description

Nigeria is the Africa׳s second largest economy, following South Africa [13]. It is clearly known that Nigeria represents 15 per cent of Africa׳s population with 11 per cent of her contribution being Africa׳s total output as well as 16 per cent of its foreign reserves, accounting for half of the population [14].

Institutional framework with a focus on the survival of informal electronics entrepreneurs’ businesses in Nigerian was the focus of this study. Specifically, the study examined the survivals of electronics informal business on the ability of the manipulative and avoidance skills of the entrepreneurs. However, emphasis was laid on the four southwestern states in Nigeria exhibiting the presence of informal electronics entrepreneurs in their state capital. The informal electronics market in this states were considered due to the high presence of clustered informal entrepreneurs relevant to the study and also because the main aim of the informal electronics businesses was to alleviate poverty while reducing unemployment. The informal entrepreneurs were given thoughtfulness because of possibility of discernments that could be provided from their perceptions on the business survival and operation admits the securing enforcement agencies.

## 3 Experimental design, materials and methods

Four states were selected from Southwest region in Nigeria. Three hundred and twenty informal entrepreneurs in the selected states participated in this study. Ethical consideration in research process was ensured. These informal entrepreneurs belong to an informal association and the researchers were given permission on the basis of anonymity i.e. the researchers should not disclose their identities. Data were gathered from informal entrepreneurs across the various markets in the selected states with the aid of a researcher- made questionnaire based on the works of [4], [5], [11], [12], [14]. The demographic data presented information based on gender. age, marital status and educational qualification. Questions relating to the prevalence of avoidance of institutional framework and business survival was considered in the questionnaire.

There was a meaningful relationship between the engagement of tactics in manipulating the institutions designed to ensure the structure of businesses are done formally and the survival of informal electronics businesses in southwest Nigeria.

The collected data were coded and entered into SPSS version 25. Data analysis was performed; using SPSS-25. Data was analyzed applying descriptive statistical tests which involved mean and standard deviations scores.

It should be noted that Statistical Package for Social Sciences may be inadequate to address some advanced modeling and development of statistical approaches however, for a simple analysis such as descriptive analysis; the key selling point is its spread-out statistics inquiry options. The selling point of these functions are its level of automation to the point that one simply needs to select the related variables and matching applications for results and analysis and the package does the rest.

## 4 Conclusion and implications of the study

The tendency for informal entrepreneurs downsizing the unemployment syndrome while contributing to the Nigerian economic society depends on the restructuring of policies that will fine-tune the business environment. This has implications on how the operations of agencies of involved institutions is regulated to ensure the existence of informal entrepreneurs. To this end, the data are widely accessible to ensure critical and comprehensive analysis.

## References

[1] Okigbo C. (1990). Gatekeeping in the Nigerian press. Afr. Media Rev..

[2] Roberts C. (2005). Gatekeeping theory: an evolution. Recuper. el.

[3] Webb J.W., Tihanyi L., Ireland R.D., Sirmon D.G. (2009). You say illegal, I say legitimate: entrepreneurship in the informal economy. Acad. Manag. Rev..

[4] Webb J.W., Bruton G.D., Tihanyi L., Ireland R.D. (2013). Research on entrepreneurship in the informal economy: framing a research agenda. J. Bus. Ventur..

[5] Adebanji A.W., Iyiola O., Ogunnaike O.O., Ibidunni S.A., Akinde O., Olubodun I. (2017). Empirical Assessment of Social Motivation and Performance of Informal Entrepreneurs in Computer Village, Lagos State.

[6] Mathias B.D., Lux S., Crook T.R., Autry C., Zaretzki R. (2015). Competing against the unknown: the impact of enabling and constraining institutions on the informal economy. J. Bus. Ethics.

[7] Portes A., Castells M., Benton L.A. (1989). The Informal Economy: Studies in Advanced and Less Developed Countries.

[8] Pathak S., Laplume A.O., Xavier-Oliveira E. (2016). Informal institutions and technology use by entrepreneurs: an empirical study across 18 emerging markets. Int. J. Emerg. Mark..

[9] Fuentelsaz L., González C., Maícas J.P., Montero J. (2015). How different formal institutions affect opportunity and necessity entrepreneurship. BRQ Bus. Res. Q..

[10] Webb J.W., Bruton G.D., Tihanyi L., Ireland R.D. (2013). Research on entrepreneurship in the informal economy: framing a research agenda. J. Bus. Ventur..

[11] Z.J. Acs, S. Desai, P. Stenholm, R. Wuebker, Institutional Drivers of Informal Entrepreneurship, 2014.

[12] Williams C.C., Nadin S. (2012). Tackling the hidden enterprise culture: government policies to support the formalization of informal entrepreneurship. Entrep. Reg. Dev..

[13] Neuwirth R. (2011). How MTN is profiting from Nigeria׳s informal economy. How We Made Afr..

[14] Sanusi L.S. (2010). Growth prospects for the Nigerian economy. Convoc. Lect. Deliv. Igbinedion Univ. Eighth Convoc. Cerem..

